# New Methodology for Estimating the Burden of Infectious Diseases in Europe

**DOI:** 10.1371/journal.pmed.1001205

**Published:** 2012-04-17

**Authors:** Mirjam Kretzschmar, Marie-Josée J. Mangen, Paulo Pinheiro, Beate Jahn, Eric M. Fèvre, Silvia Longhi, Taavi Lai, Arie H. Havelaar, Claudia Stein, Alessandro Cassini, Piotr Kramarz, for the BCoDE consortium

**Affiliations:** 1 Centre for Infectious Disease Control, National Institute for Public Health and the Environment (RIVM), Bilthoven, The Netherlands; 2 Julius Centre for Health Sciences and Primary Care, University Medical Centre Utrecht, Utrecht, The Netherlands; 3 School of Public Health, Bielefeld University, Bielefeld, Germany; 4 Institute of Public Health, Medical Decision Making and Health Technology Assessment, Department of Public Health and Health Technology Assessment, UMIT—University for Health Sciences, Medical Informatics and Technology, Hall in Tirol, Austria; 5 Division of Public Health Decision Modelling, Health Technology Assessment and Health Economics, Oncotyrol Center for Personalized Cancer Medicine, Innsbruck, Austria; 6 Centre for Immunity, Infection and Evolution, Institute for Immunology and Infection Research, School of Biological Sciences, University of Edinburgh, Edinburgh, United Kingdom; 7 Institute of Hygiene and Public Health, Catholic University of the Sacred Heart, Rome, Italy; 8 National Institute for Health Development, Tallinn, Estonia; 9 Department of Public Health, University of Tartu, Tartu, Estonia; 10 Institute for Risk Assessment Sciences, Utrecht University, Utrecht, The Netherlands; 11 Regional Office for Europe, World Health Organization, Copenhagen, Denmark; 12 European Centre for Disease Prevention and Control, Stockholm, Sweden

## Abstract

Mirjam Kretzschmar and colleagues describe the BCoDE project, which uses a pathogen-based incidence approach to better estimate the infectious disease burden in Europe.

**Summary Points**

- The major objectives of the Burden of Communicable Diseases in Europe (BCoDE) study are to further develop the methodology to estimate the burden of infectious diseases (IDs), and to estimate and report on the current and future burden of IDs in the European Union member states and European Economic Area/European Free Trade Association countries.
- The BCoDE project uses a pathogen-based incidence approach to generate estimates, fully taking into account all chronic and long-term sequelae that can be causally related to an infectious agent.
- An important focus is the assessment of underreporting and under-ascertainment in various types of incidence data.
- Future challenges are the integration of demographic changes and infection dynamics into the methodology for estimating the burden of IDs.

## Disease Burden Estimates for Infectious Diseases

Baseline comprehensive estimates of infectious disease (ID) burden are needed for effective planning and prioritizing of limited public health resources. Over the last three decades, efforts have been made to derive and apply methods to estimate disease burden at population scales. In particular, the Global Burden of Disease (GBD) project [1] has made important progress in this area methodologically and in terms of output estimates, and is based on available evidence that therefore supports health-care policy making [2]. While the incidence of IDs has in general decreased substantially in Europe over the last century, newly emerging and re-emerging IDs pose serious threats to population health [3],[4]. According to recent estimates from the GBD project, IDs represent less than 10% of the total burden of disease in Europe [5],[6]. This figure, however, might underestimate the real burden due to IDs in the European region because it does not fully take into account the whole spectrum of long-term sequelae caused by infections. Here, we outline an approach taken to adapt burden estimate methods to the European situation; the approach capitalizes on the generally good data quality in the European Union, but also takes formal, quantitative account of underreporting and under-ascertainment, as well as the burden of all important sequelae associated with an infection.

In the autumn of 2006, the Dutch National Institute for Public Health and the Environment (RIVM) conducted a pilot study on behalf of the European Centre for Disease Prevention and Control (ECDC) to illustrate the potential of the disease burden concept [2], to explore data availability and quality, and to stimulate debate [7]–[9]. In July 2009, the Burden of Communicable Diseases in Europe (BCoDE) project was launched by the ECDC with the major objectives of furthering development of the methodology to estimate the burden of IDs, and providing estimates of the current and future burden of IDs in the EU member states and European Economic Area/European Free Trade Association countries. These estimates take into account the burden of acute illness and of fatal cases, as well as of sequelae and complications associated with the infectious agent (e.g., infection-associated cancers). To do this in a consistent way, an approach was developed that attributes all burden generated by an infection with a specific pathogen to the infection event using information on disease progression. Future aims of the project are to consider the dynamic aspects of ID epidemics, the impact of public health interventions, and emerging trends like demographic change and climate change.

## Composite Health Measures for Infectious Diseases

Composite measures for disease burden were used on a global scale by the World Bank [10] and later in landmark studies of the GBD project [1],[5],[11]. Those studies estimated the global burden of a whole spectrum of diseases, including conditions as diverse as mental illness, injuries, chronic diseases, and IDs. To comprehensively present and compare the impact of these conditions on population health and mortality, composite measures of population health were developed and used to sum up the impact of adverse health events on quality of life and life expectancy in one single metric [2],[12]. The impact of every adverse event on health can be measured by the number of life years lost due to premature death and the number of life years lost due to disability. The latter requires measuring the impact of disease on quality of life using disability weights. Both the number of life years lost due to premature death and the number of life years lost due to disability are estimated by use of a reference that reflects an ideal health goal, and add up to a disability-adjusted life year (DALY) [2].

There are a number of challenges when computing the disease burden for ID. One difficulty is the fact that symptomatic as well as asymptomatic infections may lead to long-term chronic sequelae, which might therefore not always be recognized as being originally caused by an infection [13]. More generally, for many IDs the possible relationships with later chronic sequelae are not clearly established, and therefore criteria have to be specified to decide when the strength of evidence is sufficient for attributing long-term morbidity and/or mortality associated with those sequelae to their infectious cause [14].

**Figure 1:**
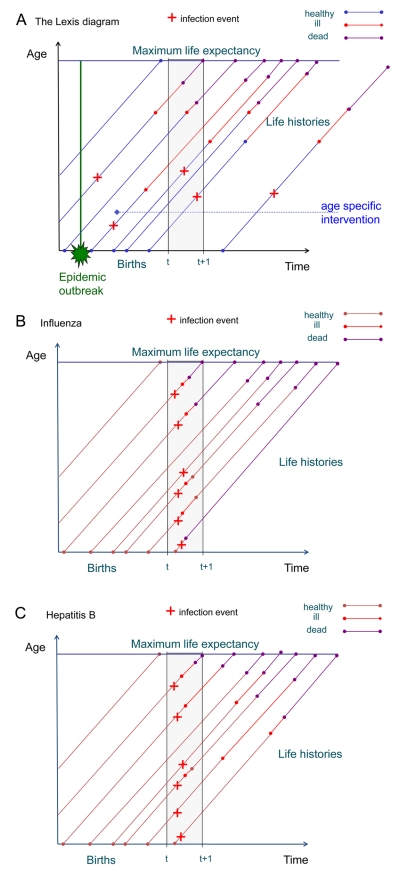
The Lexis diagram shows events by age and time. (A) This Lexis diagram shows the occurrence of infection, disease, and death in individual life histories in the time–age plane. An epidemic outbreak affects several cohorts of individuals at a specific time, but may cause disease burden at different times later on. An age-specific intervention starts at a certain time and affects all cohorts reaching the specific age from that time onward. It does not prevent disease burden from earlier infections. Incidence may cause burden within a time window of observation, but also at later times within the life histories of the affected individuals. (B) Here the Lexis diagram shows the occurrence of influenza cases within the time period of one year. All burden generated by morbidity (red) occurs also within that time period. Burden due to mortality is from deaths occurring in the same year as infection. (C) The Lexis diagram for hepatitis B shows that the burden due to morbidity is spread out over many years following the incident infections in the year starting at time *t*.

Another difficulty in estimating the burden of ID is the fact that they occur on very different time scales. While for an influenza infection acute illness and sequelae occur within a time period of weeks, for HIV infection and hepatitis B infection the time between acute infection and death may span decades. Attributing long-term sequelae to infection with a specific pathogen therefore may require adding disease burden that occurs over long time periods. This is visualized by plotting individual life trajectories in a Lexis diagram, a tool used by demographers to represent demographic processes in the time–age plane [15],[16]. The Lexis diagram shows how the incidence of infection and the resulting sequelae may be distributed in the time–age plane (Figure 1A). For an infection with only short-term symptoms and sequelae, incidence and sequelae lie within a well-defined time slice in the plane, whereas for infections with long-term sequelae or late onset of sequelae, these are distributed over a larger area outside the time slice under consideration (Figure 1B and 1C). In a steady state situation this is not a problem, but if there are temporal fluctuations in incidence, interpretation and comparison of disease burdens is more intricate.

## Pathogen-Based Incidence Approach

In the first phase of the BCoDE project, the disease burden was estimated for four countries (Estonia, Germany, Italy, and The Netherlands) and 32 IDs (Table 1). The diseases included in the BCoDE study were selected from a list of 49 IDs that fall under the mandate of the ECDC as part of the network for epidemiological surveillance and control of communicable diseases in the European Union and European Economic Area/European Free Trade Association states [17]. For the selection, a list of criteria was applied that assessed the importance of an ID and the potential difficulties in estimating the burden (e.g., the availability of disability weights) [18]. While some nosocomial pathogens are on the list for future burden estimates, their estimation was postponed to a later stage of the project because they require methods to deal with co-morbidity and are less amenable to the pathogen-based approach.

**Table 1:** Infectious diseases for which burden estimates were derived in the BCoDE project.

Disease Group	Infectious Disease
Respiratory infections	Seasonal influenza
	Legionellosis
	Tuberculosis
Sexually transmitted infections	Chlamydia
	Gonococcal infections
	Hepatitis B
	Hepatitis C
	HIV
	Syphilis
Food- and waterborne infections	Campylobacteriosis
	Cryptosporidiosis
	Infection with STEC/VTEC
	Giardiasis
	Hepatitis A
	Listeriosis
	Salmonellosis
	Shigellosis
	Toxoplasmosis
	Creutzfeldt-Jakob disease
Zoonotic and vectorborne infections	Q fever
	Tick-borne encephalitis
Vaccine-preventable infections	Diphtheria
	Invasive haemophilus influenzae disease
	Invasive pneumococcal infections
	Measles
	Invasive meningococcal disease
	Mumps
	Pertussis
	Poliomyelitis
	Rabies
	Rubella
	Tetanus

STEC/VTEC, shigatoxin-producing *E. coli*/verocytotoxin-producing *E. coli*.

**Figure 2:**
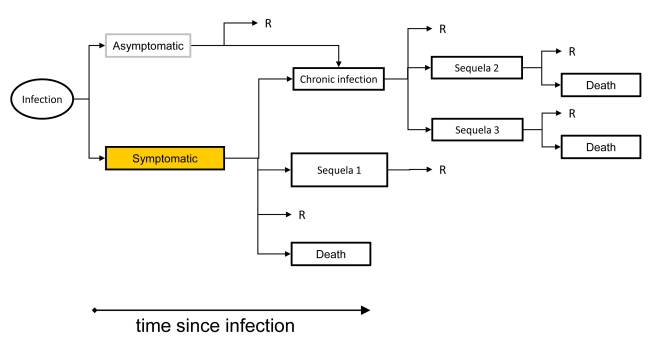
An outcome tree linking exposure, infection and all sequelae. The outcome tree displays how individuals may progress through various stages of infection, disease, and death. The process can be quantified by attaching proportions to the arrows depicting transitions, and durations to the various health outcomes. “R” denotes full recovery from infection and/or disease.

The aim of the pilot study was to gain experience with the new methodological approach and to assess data availability and quality. We obtained notification data and other surveillance data from national public health institutes, performed literature reviews to extract information about disease progression and underreporting, and developed outcome trees for all IDs included in the study [18]. Computational models were developed for estimating the burden in a standardized manner. The disease burden was calculated as DALYs stratified by age and sex. We used GBD disability weights where available, and weights from other published studies otherwise [19]. We will update those weights when new GBD disability weights become available [20]. We calculated DALYs using a pathogen-based incidence approach, which links sequelae to their infectious cause [7]. In this approach, the incidence of infections from a specific pathogen in a particular year is linked to all related health outcomes through an outcome tree or disease progression model (Figure 2). An outcome tree gives a qualitative representation of the progression of disease in time by ordering all relevant health outcomes following infection and illustrating their conditional dependency. To derive quantitative estimates using an outcome tree, information on the probability of entering and the time spent in each health state was required. This information was extracted from published literature and validated by expert consultation. Then, using the incidence of an infection in a given year as a starting point, and based on knowledge of the expected frequency of health outcomes following from the infection, the burden of an ID was estimated.

The BCoDE project relies heavily on notification data and on other readily available data from surveillance systems, which are relatively well developed in the European context. We based our estimates mainly on three types of data obtained from different surveillance sources: incidence of symptomatic infections (e.g., notification data or data from lab surveillance), incidence of hospitalized cases of infection (hospitalization data), and incidence of death due to the infection (cause of death data from vital registration systems).

Data from routine surveillance sources need to be adjusted to correct for under-reporting and under-ascertainment when estimating disease burden from those data. Under-ascertainment refers to cases or exposures in the community in individuals who never seek health care and are therefore not registered in any notification or surveillance system. Underreporting refers more specifically to cases in individuals who seek health care but whose infection status is misdiagnosed or misclassified, and whose infection details are therefore not passed on to national surveillance systems.

Multiplication factors were applied to the reported numbers of cases of a particular disease in order to estimate the true numbers of cases. A systematic method for estimating these was developed, which will be fully reported elsewhere. Briefly, multiplication factors were developed by comparing incidence or exposure in the general population (preferentially determined by community-based or serological studies) with notified case data (including incidence of hospitalizations, laboratory-confirmed cases, general practitioner cases, and deaths attributable to the disease). Multiplication factors are disease-specific since the amount of under-reporting varies by disease. Ideally, they should also be country-specific (owing to variations in disease exposure, health-care systems, and availability of treatment, as well as cultural, social, and technological differences) and age- and sex-specific. In some infections, like influenza, even seasonal strains will cause a varying degree of symptomatic disease and associated health-seeking behavior [21]. However, we did not have such detailed information available; in most cases, we had only rough estimates for the ratio of reported to unreported cases.

**Figure 3:**
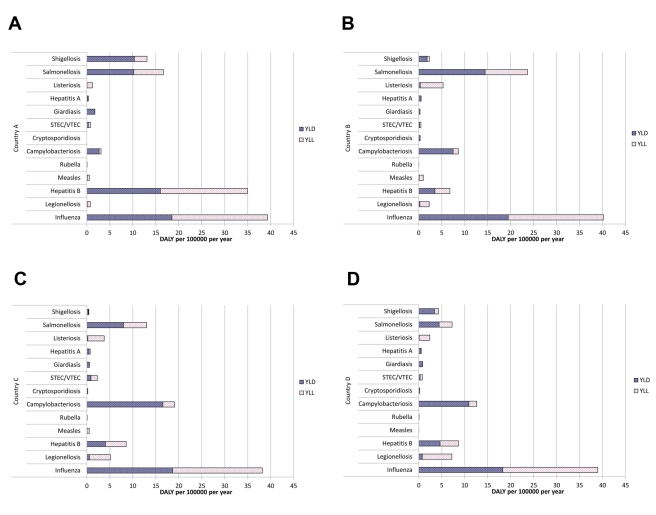
Preliminary estimates of the burden of disease in terms of DALYs per 100,000 individuals per year for selected infections in four European countries. The differences seen between countries (A–D) may be due to differences in surveillance and/or to differences in the (distribution of) incidence of infections in populations. STEC/VTEC, shigatoxin-producing *Escherichia coli*/verocytotoxin-producing *E. coli*; YLD, number of life years lost due to disability; YLL, number of life years lost due to premature death.

Based on health outcomes defined in outcome trees of IDs, we collected incidence data for acute illness and other health outcomes, if available. For each health outcome, incidence data (morbidity and mortality) were collected for a three-year period (1 January 2005–31 December 2007) and used as input into estimation models. These years were chosen because the ECDC had established standards for case reporting on the European level, and data collection was completed at the time the project started. For computing the estimates, data on incidence of acute illness were preferentially used, while data on incidence of other health outcomes were used for validation. If the incidence of acute infections was not available, incidence of morbidity or mortality was used. Based on the three study years, a crude annual mean incidence was estimated, stratified by age (in five-year classes) and sex. Where necessary, these incidences were adjusted by factors correcting for underreporting and under-ascertainment. For sequelae, but also for other health outcomes where no incidence data were available, we estimated the number of cases using the probability of the occurrence of outcomes, taking into consideration the conditional dependency of the different health outcomes as defined by the outcome tree. Preliminary results are shown in Figure 3 (see also [22],[23]); final results will be published elsewhere.

## Future Challenges and Conclusions

The dynamics of ID transmission occurs in widely differing time scales depending on the pathogen. Clearly, infections that spread on the time scale of the average generation time of a population will be closely linked to changes in demography, social and behavioral changes, and the implementation of preventive measures. ID can influence a population's demography by affecting mortality and therefore average life expectancy, or by influencing fertility rates. On the other hand, demography also influences the transmission of ID by determining the relative sizes of susceptible and vulnerable populations. [24]. While a severe impact of ID on the demography of entire nations has been observed in developing countries, in the industrialized world the aging of populations may have an impact on the burden of ID [25]–[27]. Prevention programs such as mass vaccination tend to increase the average age at which an exposure to infection takes place and therefore increase the probability of severe complications for some diseases. For some IDs, reactivation of latent infections acquired at a young age may occur at an older age because of changes in the functioning of the immune system. Finally, demographic flow leads to shifts in the immune status of entire populations, possibly resulting in increasing risks of large outbreaks in vulnerable population groups.

At present the methods used in burden of disease calculations rest on steady state assumptions regarding demography and epidemiology. However, there are few IDs for which the epidemiological situation has remained even remotely stable over the time span of the last 50 years, not only because of the implementation of large-scale prevention programs, but also because of enormous changes in mobility patterns and life styles [28]. Also, population densities have increased, and migration is increasingly important in determining a country's epidemiological situation. In addition, it is expected that climate change will have a major impact on the distribution of IDs within the coming century [29]. In Europe, awareness is increasing that pathogens that have been limited to more tropical climates may cause major outbreaks or even become endemic in countries of the temperate climate zone. Also, changes in agricultural production systems, urbanization, and changing contact patterns with animals lead to increasing risks of zoonotic infections or emerging infections of zoonotic origin. These diseases may not contribute much to the overall burden at present, but we would like to anticipate the future burden that Europe might be facing if these diseases continue to emerge on a larger scale [30].

We used a pathogen-based incidence approach that attributes all burden generated by an infection to the time of incidence of the infection. While this has the advantage of consistently attributing the burden to its infectious cause, the approach also has some serious limitations. For many pathogens that cause broad, nonspecific disease syndromes, it is difficult to attribute morbidity to a specific pathogen. Conditions like diarrhea, pneumonia, or encephalitis may be caused by many different pathogens, and usually we do not have the specific information to attribute morbidity to specific pathogens. Even if such information is available, a (large) proportion of cases may not be attributable to any specific pathogen. Also, co-morbidity and co-infections may play an important role, especially in hospital settings. One strategy to deal with these limitations may be to use information on the occurrence of syndromes as a validation tool for estimates derived from the pathogen-based approach. These limitations highlight the need for further research in this area.

Recent advances in mathematical and statistical methods for studying IDs will provide new tools for future disease burden estimation. Dynamic transmission models—already widely used for the analysis of epidemiological data and the effects of intervention—will be used to describe temporal dynamics of outbreaks and the impact of large-scale intervention measures [31]. These models will be combined with models from mathematical demography [16] to account for changes in population age structure and life expectancy. Bayesian statistical methods for parameter estimation provide tools for combining data from various sources into a consistent estimate, allowing the weighting of evidence according to its perceived reliability [32]. Combining dynamic transmission models that include demographic modeling with Bayesian estimation methods will be the methodological toolkit for future burden estimates for ID within the BCoDE project. A toolkit for the application of burden estimation models, which is currently being developed by the consortium, will soon be available for public health policy makers, to support national disease burden studies of IDs.

## Supporting Information

Alternative Language Abstract S1 **Estonian translation of the summary by T. L.**
(PDF)

Alternative Language Abstract S2 **German translation of the summary by B. J.**
(PDF)

Alternative Language Abstract S3 **Italian translation of the summary by S. L.**
(PDF)

## Abbreviations

BCoDE: Burden of Communicable Diseases in Europe
DALY: disability-adjusted life year
ECDC: European Centre for Disease Prevention and Control
GBD: Global Burden of Disease
ID: infectious disease

## References

[1] Murray CJ, Lopez AD (1997). Global mortality, disability, and the contribution of risk factors: Global Burden of Disease Study.. Lancet.

[2] Murray CJ, Salomon JA, Mathers C, Lopez AD (2002). Summary measures of population health.

[3] Morens DM, Folkers GK, Fauci AS (2004). The challenge of emerging and re-emerging infectious diseases.. Nature.

[4] Jones KE, Patel NG, Levy MA, Storeygard A, Balk D (2008). Global trends in emerging infectious diseases.. Nature.

[5] Lopez AD, Mathers CD, Ezzati M, Jamison DT, Murray CJ (2006). Global and regional burden of disease and risk factors, 2001: systematic analysis of population health data.. Lancet.

[6] Pinheiro P, Mathers CD, Krämer A, Krämer A, Kretzschmar M, Krickeberg K (2010). The global burden of infectious diseases.. Modern infectious disease epidemiology.

[7] van Lier EA, Havelaar AH (2007). Disease burden of infectious diseases in Europe: a pilot study. RIVM report 215011001.

[8] van Lier EA, Havelaar AH, Nanda A (2007). The burden of infectious diseases in Europe: a pilot study.. Euro Surveill.

[9] Jakab Z (2007). Why a burden of disease study?. Euro Surveill.

[10] The World Bank (1993). World development report 1993.

[11] Lopez AD, Mathers CD (2006). Measuring the global burden of disease and epidemiological transitions: 2002–2030.. Ann Trop Med Parasitol.

[12] Murray CJL (1994). Quantifying the burden of disease: the technical basis for disability- adjusted life years.. Bull World Health Organ.

[13] Zou S (2001). Applying DALYs to the burden of infectious diseases.. Bull World Health Organ.

[14] Franco EL, Correa P, Santella RM, Wu X, Goodman SN (2004). Role and limitations of epidemiology in establishing a causal association.. Semin Cancer Biol.

[15] Keiding N (1990). Statistical inference in the lexis diagram.. Philos Trans Phys Sci Eng.

[16] Keyfitz N, Caswell H (2005). Applied mathematical demography.

[17] European Parliament (1998). Decision No 2119/98/EC of the European Parliament and of the Council of 24 September 1998 setting up a network for the epidemiological surveillance and control of communicable diseases in the Community.. http://eur-lex.europa.eu/LexUriServ/LexUriServ.do?uri=CELEX:31998D2119:EN:NOT.

[18] European Centre for Disease Prevention and Control (2010). Methodology protocol for estimating burden of communicable diseases.

[19] Stouthard MEA, Essink-Bot M-L, Bonsel GJ, on behalf of the Dutch Disability Weights Group (2000). Disability weights for diseases: a modified protocol and results for a Western European region.. Eur J Public Health.

[20] Salomon JA (2010). New disability weights for the global burden of disease.. Bull World Health Organ.

[21] van den Wijngaard CC, van Asten L, Meijer A, van Pelt W, Nagelkerke NJ (2010). Detection of excess influenza severity: associating respiratory hospitalization and mortality data with reports of influenza-like illness by primary care physicians.. Am J Public Health.

[22] Plass D, Pinheiro P, Kraemer A, Gibbons C, Mangen MJJ (2011). Burden of infectious diseases in Germany—preliminary results from the Burden of Communicable Diseases in Europe (BCoDE) pilot study [abstract]..

[23] Bijkerk P, van Lier A, Swaan C, Kretzschmar M (2011). Staat van Infectieziekten in Nederland, 2010. RIVM report 210211007.

[24] Manfredi P, Williams JR (2004). Realistic population dynamics in epidemiological models: the impact of population decline on the dynamics of childhood infectious diseases. Measles in Italy as an example.. Math Biosci.

[25] Bijkerk P, van Lier EA, van Vliet JA, Kretzschmar ME (2010). [Effects of ageing on infectious disease].. Ned Tijdschr Geneeskd.

[26] Liang SY, Mackowiak PA (2007). Infections in the elderly.. Clin Geriatr Med.

[27] Jarrett PG, Rockwood K, Carver D, Stolee P, Cosway S (1995). Illness presentation in elderly patients.. Arch Intern Med.

[28] Cliff A, Haggett P (2004). Time, travel and infection.. Br Med Bull.

[29] Campbell-Lendrum D, Woodruff R (2006). Comparative risk assessment of the burden of disease from climate change.. Environ Health Perspect.

[30] Havelaar AH, van Rosse F, Bucura C, Toetenel MA, Haagsma JA (2010). Prioritizing emerging zoonoses in the Netherlands.. PLoS ONE.

[31] Jit M, Brisson M (2011). Modelling the epidemiology of infectious diseases for decision analysis: a primer.. Pharmacoeconomics.

[32] Sweeting MJ, De Angelis D, Hickman M, Ades AE (2008). Estimating hepatitis C prevalence in England and Wales by synthesizing evidence from multiple data sources. Assessing data conflict and model fit.. Biostatistics.

