# Supplementary material for: New Methodology for Estimating the Burden of Infectious Diseases in Europe
Source: PLoS Med. 2012 Apr 17;9(4):e1001205. doi: 10.1371/journal.pmed.1001205 (PMC3328443; doi:10.1371/journal.pmed.1001205)
Supplement: Alternative Language Abstract S1 — Estonian translation of the summary by T. L. (PDF) [file pmed.1001205.s001.pdf]

Translation of the summary into Estonian by Taavi Lai

- Projekti „Nakkushaigustest tingitud tervisekaotus Euroopas“ („*Burden of Communicable Diseases in Europe*“ e. BCoDE) peamisteks eesmärkideks on nakkushaigustest tingitud haiguskoormuse arvutamise metoodika jätkuv arendamine ja neist haigustest põhjustatud tänase ning tulevase tervisekaotuse leidmine Euroopa Liidu (EL) ja Euroopa Majanduspiirkonna ning Euroopa Vabakaubanduse Assotsiatsiooni (EEA/EFTA) riikides.

- BCoDE projektis kasutatakse nakkushaigustest tingitud tervisekaotuse leidmiseks haigustekitajate ning esmashaigestumise põhist lähenemist. Selline lähenemine võimaldab täiel määral arvesse võtta kõiki võimalikke kroonilisi ja pikaajalisi nakkushaigustest tingitud järelmeid ning terviseseisundeid.

- BCoDE projektis on suure tähelepanu all ka nakkushaiguste alatuvastamise ja – raporteerimise suuruse ja mõju hindamine kasutatavates esmashaigestumise andmetes.

- Järgnevateks lahendamist vajavateks väljakutseteks on rahvastiku ja nakkushaiguste dünaamika mõjude arvesse võtmine nakkushaiguste tervisekaotuse hindamiseks kasutatavates mudelites.
