# Supplementary material for: New Methodology for Estimating the Burden of Infectious Diseases in Europe
Source: PLoS Med. 2012 Apr 17;9(4):e1001205. doi: 10.1371/journal.pmed.1001205 (PMC3328443; doi:10.1371/journal.pmed.1001205)
Supplement: Alternative Language Abstract S2 — German translation of the summary by B. J. (PDF) [file pmed.1001205.s002.pdf]

Translation of the summary into German by Beate Jahn

- Die Studie über die Krankheitslast übertragbarer Krankheiten in Europa (Burden of Communicable Diseases in Europe -BCoDE) verfolgt das Ziel eine Methodologie zu entwickeln für die Abschätzung der Krankheitslast von Infektionskrankheiten und der Schätzung und Dokumentation derzeitiger und zukünftiger Krankheitslast von Infektionskrankheiten in den EU MS und EEA/EFTA Staaten.

- Im Rahmen des BoCDE Projekts wird ein pathogenbasierter Inzidenzansatz für die Schätzung verwendet, so dass alle chronischen Folgeerkrankungen und Spätschäden, die in ursächlichem Zusammenhang mit dem Infektionserreger stehen, berücksichtigt werden können.

- Ein wichtiger Schwerpunkt des BCoDE Projekts ist die Beurteilung des Umfangs von unvollständiger Diagnose und Erfassung bei den verschiedenen Arten von Inzidenzdaten.

- Eine weitere Herausforderungen hinsichtlich der Methodologie zur Abschätzung der Krankheitslast von Infektionskrankheiten stellt die Berücksichtigung demografischer Veränderungen und der Infektionsdynamik dar.
