# Supplementary material for: New Methodology for Estimating the Burden of Infectious Diseases in Europe
Source: PLoS Med. 2012 Apr 17;9(4):e1001205. doi: 10.1371/journal.pmed.1001205 (PMC3328443; doi:10.1371/journal.pmed.1001205)
Supplement: Alternative Language Abstract S3 — Italian translation of the summary by S. L. (PDF) [file pmed.1001205.s003.pdf]

Translation of the summary into Italian by Silvia Longhi

- Gli obiettivi principali dello studio sul “Carico delle malattie trasmissibili in Europa“ (Burden of Communicable Diseases in Europe -BCoDE) sono lo sviluppo e il perfezionamento della metodologia per l’analisi del burden delle malattie infettive e la sua applicazione per la stima del carico di malattia attuale e futuro negli Stati membri dell’UE e nei Paesi EEA/EFTA.

- Il progetto BCoDE utilizza un approccio “pathogen-based” in modo da generare stime che tengano conto anche delle complicanze e delle sequele di lungo termine associate all’agente infettivo.

- Un aspetto cruciale del progetto BCoDE riguarda la stima della sottodiagnosi e della sottonotifica dei dati di incidenza.

- Una sfida per il futuro consisterà nell’integrazione dei cambiamenti demografici e della dinamica delle infezioni nella metodologia utilizzata per la stima del carico delle malattie infettive.
